# Reconstitution of Membrane Proteins into Model Membranes: Seeking Better Ways to Retain Protein Activities

**DOI:** 10.3390/ijms14011589

**Published:** 2013-01-14

**Authors:** Hsin-Hui Shen, Trevor Lithgow, Lisandra L. Martin

**Affiliations:** 1 Department of Biochemistry and Molecular Biology, Monash University, Melbourne 3800, Australia; E-Mail: trevor.lithgow@monash.edu; 2 School of Chemistry, Monash University, Clayton, VIC 3800, Australia; E-Mail: lisa.martin@monash.edu

**Keywords:** nanodiscs, liposomes, supported lipid bilayer, monolayer, membrane protein activity

## Abstract

The function of any given biological membrane is determined largely by the specific set of integral membrane proteins embedded in it, and the peripheral membrane proteins attached to the membrane surface. The activity of these proteins, in turn, can be modulated by the phospholipid composition of the membrane. The reconstitution of membrane proteins into a model membrane allows investigation of individual features and activities of a given cell membrane component. However, the activity of membrane proteins is often difficult to sustain following reconstitution, since the composition of the model phospholipid bilayer differs from that of the native cell membrane. This review will discuss the reconstitution of membrane protein activities in four different types of model membrane—monolayers, supported lipid bilayers, liposomes and nanodiscs, comparing their advantages in membrane protein reconstitution. Variation in the surrounding model environments for these four different types of membrane layer can affect the three-dimensional structure of reconstituted proteins and may possibly lead to loss of the proteins activity. We also discuss examples where the same membrane proteins have been successfully reconstituted into two or more model membrane systems with comparison of the observed activity in each system. Understanding of the behavioral changes for proteins in model membrane systems after membrane reconstitution is often a prerequisite to protein research. It is essential to find better solutions for retaining membrane protein activities for measurement and characterization *in vitro*.

## 1. Introduction

The cell membrane separates intracellular components from the outside environment and is constituted by various phospholipids, cholesterol, glycolipids and proteins. Integral membrane proteins have at least one polypeptide segment spanning the membrane bilayer whereas peripheral membrane proteins are temporarily attached to the lipid bilayer or to integral membrane proteins by various interactions such as hydrophobic, electrostatic and other types of non-covalent interactions. Membrane proteins work as a selective filter to regulate molecules entering cells and also serve in communicating with the surrounding environment. Thus, membrane proteins play an essential role in the physiological functions needed for cell survival.

The functional activities of membrane proteins are modulated by the structure of the surrounding lipids molecules in the membrane [1,2]; thus the composition of the lipid bilayer can affect the inter- or intra-molecular interactions between the lipid bilayer and membrane proteins [3]. Investigating membrane proteins *in vivo* is difficult because the membrane proteins are associated with a complex mixture of other proteins, and are prone to aggregation in solution [4]. It is still a major challenge at this stage to extract information needed *in vivo* to address specific questions in the function of the cell membrane.

**Figure 1:**
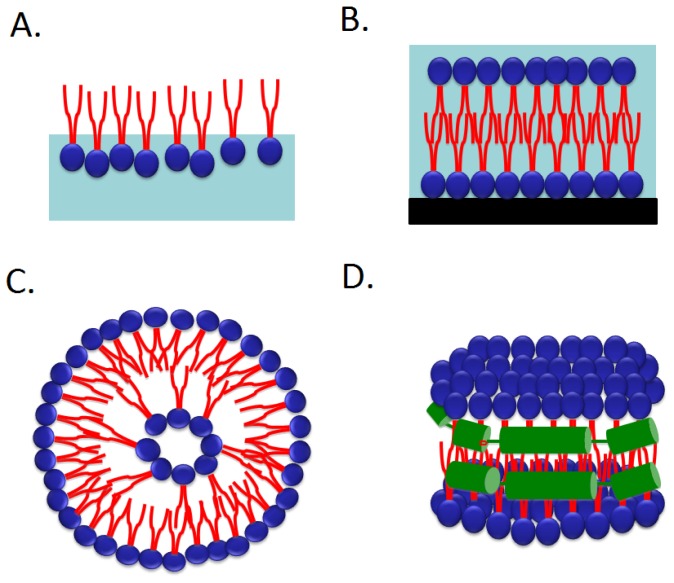
Schematic drawings of (**A**) monolayer, (**B**) supported lipid bilayer, (**C**) liposomes and (**D**) nanodisc. Phospholipids contain two fatty acid tails, shown in red and a hydrophilic head group, shown in blue. Light blue (**A** & **B**) and black in B represent water and a substrate respectively. Nanodiscs contain membrane scaffold proteins, shown in green.

To simplify cell membrane systems, model membranes such as monolayers, bilayers, liposomes and nanodiscs have been developed, enabling detailed investigation of membrane protein structure in lipid membranes. Model membrane environments more closely resemble the natural lipid bilayer than alternatives such as detergents. However, many features of phospholipid structure need to be considered and optimized in the creation of a suitable model membrane. For example, the hydrophobicity of the lipid chain defined by the lengths of the fatty acid chains, is an important parameter for retaining protein activity. Other factors affecting the reconstituted membrane protein activity are the chemical properties of the lipid head groups which control membrane hydrophilicity. Both parameters are crucial in stabilizing membrane protein structure. There are a number of approaches used to create a model membrane in order to mimic properties of the native cell membrane, and we will review these various approaches for reconstituting membrane proteins into different types of model membrane—monolayers [5], supported planar lipid bilayer [6] and liposomes [7] as shown in Figure 1A–C. We will also discuss the emerging technology of nanodiscs [8] (Figure 1D). Nanodiscs are a new class of model membrane, with attractive properties that address shortcomings of other approaches in the study of membrane proteins. The first section gives a brief summary of each method and a comparison of their strengths and weaknesses. In the following section, we describe four case studies and will compare the protein activity changes when the membrane proteins are reconstituted into different model membranes. In these case studies, we demonstrate how protein activities are modulated by the lipid environment and discuss how this environment helps to retain protein activities.

## 2. Reconstitution of Membrane Protein into Model Membranes

### 2.1. Langmuir Monolayer at the Air–Water Interface

One of the most common approaches to study the membrane protein structure and activity uses a Langmuir monolayer at the air–water interface. This method has been extensively used for more than a century [9,10]. Reconstitution of membrane proteins helps obtain further information on their organization and structure in the Langmuir membrane [11,12]. It is a simple method to create a phospholipid monolayer at an air–water interface. Basically, a desired amount of lipid or lipid mixtures are dissolved in organic solvents such as chloroform or chloroform/ethanol mixtures, followed by spreading the lipid/solvent mixtures on the water surface. By evaporating out the solvent, the phospholipid molecules self-assemble vertically as a monolayer film at the air–water interface, with their hydrophilic head groups immersed in the water and their hydrophobic tail pointed to the air as shown in Figure 1A [13]. A major advantage of using the Langmuir monolayer system is that parameters such as thickness, surface pressure, molecular area and subphase thickness can be well controlled [10]. More advanced characterization techniques, such as Π-A isotherm UV-vis adsorption, X-ray reflectivity, ellipsometry and rheology, have been developed to gain detailed information on the binding of proteins onto the phospholipid monolayer and to monitor enzyme activities when binding to the monolayer [14]. However, a limitation of Langmuir monolayers is that the lack of a layer comparing to the natural cell structure (bilayer) and the high surface tension of water that can cause protein denaturation. Despite this limitation, there are several successful studies using this approach. Two types of membrane proteins in monolayer model membrane system will be briefly described below:

#### 2.1.1. Transmembrane Protein Structure in Monolayers

Rhodopsin [15,16], bacteriorhodopsin [17,18] and the aliphatic peptide gramicidin [19,20] have been successfully reconstituted and studied in monolayers at the air–water interface. In order to obtain information on the secondary structure and orientation, the protein layer can be investigated *in situ* at the air–water interface by either polarization modulation infrared reflection absorption spectroscopy (PM-IRRAS) or X-ray reflectivity in combination with surface pressure-area isotherms [21]. The study of gramicidin is an example of such an approach, and while gramicidin is unfolded at high molecular area (low pressure), it is refolded upon compression and retains its precise structure and orientation. Likewise, for both rhodopsin and bacteriorhodopsin, the secondary structures measured in monolayers are indistinguishable from that in native membranes when appropriate conditions are used. While some experiments have suggested that spreading of rhodopsin in certain conditions (>5 m/N) leads to denaturation [21], bacteriorhodopsin, in contrast, is very stable in most testing conditions (compression and temperature change). The different properties of the protein are probably due to the ability of baceriorhodopsin to form a stable two-dimensional crystalline structure at the air–water interface [21].

#### 2.1.2. Binding of Peripheral Proteins onto Monolayer

**Figure 2:**
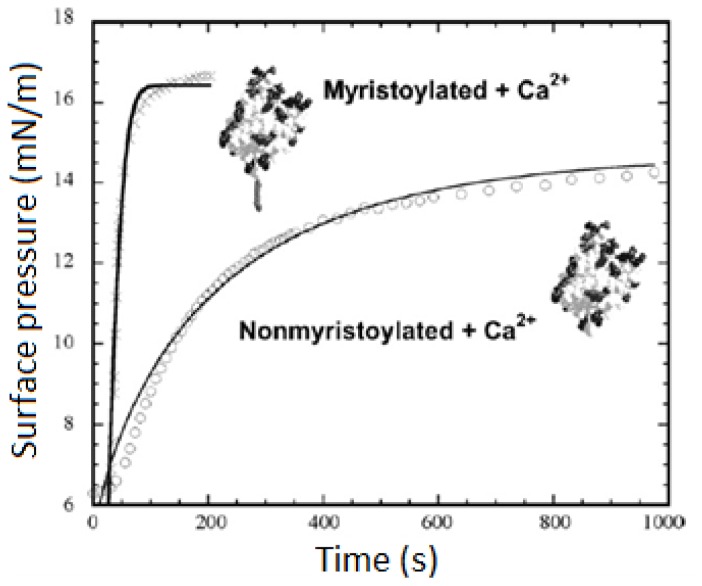
*Π*–*t* adsorption isotherms of myristoylated and nonmyristoylated recoverin adsorb onto a dimyristoyl phosphatidylcholine monolayer [21].

Phospholipid monolayers are simple model membrane systems that are perfectly suited to study the binding of peripheral proteins onto a membrane surface. Peripheral membrane proteins spontaneously bind onto phospholipid monolayers at the air–water interface by injecting themselves into the subphase underneath the lipid monolayer. In most cases, useful information can be obtained by measuring the binding of peripheral proteins onto the monolayer. For example, the kinetics and dynamics of adsorption of myristoylated and nonmyristoylated recoverin onto phospholipid monolayers have been investigated using surface pressure isotherm described in Figure 2 [21]. The curve can be fitted with stretched exponential which can convert into the rate of adsorption of myristoylated and nonmyristoylated which is 0.028 s^−1^ and 0.0048 s^−1^, respectively. This indicates that the adsorption of myristoylated Recoverin is six times faster than nonmyristoylated recoverin.

Reconstituting enzymes into the Langmuir monolayers at the air–water interface has been found to be a very useful approach to understand the hydrolysis of membrane phospholipids. For example, the interfacial recognition and adsorption of phospholipases A2 (PLA2) and phospholipases C (PLC) to the phospholipid membrane interface are poorly understood. By using this approach, it appears that both PLA2 and PLC are active at the monolayer model membrane, indicating that the kinetics of phospholipid hydrolysis at the air–water interface can be monitored by biophysical characterization techniques *in situ* such as PM-IRRAS and infrared reflection adsorption spectroscopy [22]. Moreover, it has been found that in the presence of calcium, phospholipid hydrolysis by PLA2 resulted in the production of calcium–palmitate complexes. This suggests that calcium is necessary for PLA2 secretion.

### 2.2. Supported Planar Lipid Bilayer

The formation of a supported lipid bilayer on a solid substrate was reported by Tamm and McConnell in 1985 as a new model membrane system to study the physical properties of biological membranes and their constituent lipid and protein molecules [23,24]. Supported planar lipid bilayers are prepared by several methods [25,26]. Vesicle fusion is the simplest method for supported bilayer formation and the fusion mechanism on a hydrophilic support is well understood [27,28]. Essentially, the bilayer is prepared by the fusion of small unilamellar vesicles on solid supports such as SiO_2_, glass and modified gold surface by van der Waals, electrostatic, hydration and steric forces. The supported lipid bilayer has polar hydrophilic headgroups facing the aqueous surroundings and two hydrophobic tails that face the interior of the membrane which more closely resembles biological membranes than the Langmuir monolayer. The supported lipid bilayer can confer many key functions to biological membranes. However, one side of the hydrophilic head group is still tightly attached to the solid support and this may, in some cases, affect the fluidity of the model membrane. This matters, since integral membrane proteins may not diffuse in the plane of the membrane. Furthermore the orientation of membrane proteins cannot be controlled in the supported planer lipid bilayer. To alleviate some of these problems, a new tethered polymer-supported planar lipid bilayer system was developed to investigate the reconstitution of integral membrane proteins in a laterally mobile form into the supported lipid bilayer [29].

**Figure 3:**
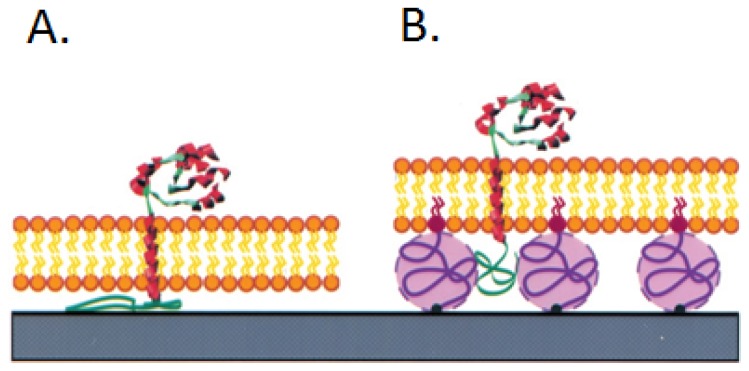
(**A**) The reconstitution of integral membrane proteins into supported lipid bilayer. (**B**) Polymer-supported lipid bilayers are designed to space the lipid bilayer from the substrate [30]. Purple represents the polymer cushions which are assembled on a substrate, shown in blue.

Wagner and Tamm [30] have successfully designed a supported lipid bilayer on a polyethyleneglycol cushion shown in Figure 3. The polymer cushion minimizes the interactions of the proteins with the substrate and the polymer. It also provides a soft support and, for increased stability, covalent linkage of the membranes to the supporting quartz or glass substrates. In low polyethyleneglycol concentration regimes, the bilayers were assembled with high lateral lipid diffusion coefficients (0.8–1.2 × 10^−8^ cm^2^/s). Cytochrome *b*5 and annexin V were used to test the polyethyleneglycol cushion system. Two populations of laterally mobile proteins were observed in the polyethyleneglycol cushion-supported bilayers. Approximately a quarter of cytochrome *b*5 diffused with a diffusion coefficient of 0.8–1.2 × 10^−8^ cm^2^/s, and more than half of the cytochrome *b*5 diffused with a diffusion coefficient of ~2 × 10^−10^ cm^2^/s. Similar results were found in the annexin V system. Annexin V diffused with two populations with diffusion coefficients of 3 × 10^−9^ cm^2^/s and 4 × 10^−10^ cm^2^/s. The new polymer-supported lipid bilayer system has increased the mobile fraction and retained the full lateral mobility of both cytochromes *b*5 and annexin when integrated or bound to the supported lipid.

Although polymer cushions allow for successful integration of small membrane proteins into bilayers, further challenges stem from studies with large transmembrane proteins. Polymer cushions cannot provide large transmembrane proteins with good solvent accessibility, or enough space for the motion; required for the activity. While several types of polymer cushions have been developed, including polymethyl methacrylate diblock polymer cushions [31], poly(ethylene imine) [32,33] cushions and poly(ethylene glycol) tethered lipopolymers [30], these cushions are mostly limited to a thickness of up to 10 nm. A recent development of a maleic anhydride copolymer thin film has film thickness up to 60 nm [34]. The hydrophilic polymer-cushioned supported lipid bilayers provide a higher mobility and homogeneous distribution of the incorporated beta-amyloid precursor protein cleaving enzyme (BACE) on the bilayer surface, and enhances the enzymatic activity of BACE (increased from 8% to 16%). Even so, the activity of the incorporated BACE remains significantly lower (16%) than that of the native enzyme (100%).

Another important classic category of membrane proteins are the transporters of ions and small molecules. Studies of how ion channels regulate the transport of substrates [7] are important for fundamental biology. However, it is challenging to incorporate ion channels in supported lipid bilayers due to leakage or instability issues. Detailed studies of ion channel conduction or gating require considerable period of time (possibly >1 h), and it is difficult to set up a stable and electrically quiet environment for the ion channel in planar lipid bilayer. A better alternative has proven to be reconstitution of ion channels into proteoliposomes.

### 2.3. Liposomes

Lipid vesicles, also known as liposomes, consist of a self-closed lipid bilayer. They have been widely used for more than 30 years to reconstitute the membrane proteins in unilamellar phospholipid vesicles. Liposomes are relatively easy to construct by procedures such as extrusion method or ultrasonication, with reverse-phase evaporation. Furthermore, giant vesicles of unilamellar or multilamellar nature can be “micro-manipulated” under an optical microscope. Reconstitution of membrane proteins in liposomes usually requires detergents wherein purified membrane proteins are solubilized in detergent, then mixed with the desired phospholipid vesicles forming an isotropic solution of mixed phospholipid-protein-detergent micelles. The detergent can then be removed slowly by dialysis, gel filtration or Biobead adsorption. When the detergent concentration reaches a critical level, the protein will spontaneously associate with the phospholipid membrane to form biologically active liposomes, called proteoliposomes. However, it has been a hard feat to control the final orientation of protein in the proteoliposomes [35], as well as the amount of protein inserted due to the limited area available. In many cases, disorientation of the protein causes aggregation. Despite these difficulties, there have been many successful cases of membrane proteins reconstituted in the proteoliposomes, and we describe two examples below.

#### 2.3.1. Activity of Membrane-Bound Enzymes

Several integral membrane proteins have been successfully reconstituted into proteoliposomes such as rhodopsin [36], G proteins [37,38], proapoptotic Bcl-2 proteins and *t*-Bid [39], phosphocholine cytidylyltransferase (CT) [40] and P protein kinase C (PKC) [41]. However, these studies also found that the resulting protein activities are sensitive to the membrane curvature of the liposomes. This indicates that different phospholipids can cause considerable curvature stress changes in the liposomes [42]. Specifically, the curvature stress has been suggested to modulate the free energy and folding of the integral membrane proteins [43]. Sometimes the activity of different enzymes is modulated by the same driving force of the membrane curvature, but there may also be variation of activity through different mechanisms. For example, the activities of both CT [40] and PKC [41] are enhanced by increasing the negative curvature strain of the membrane. The activity of CT appears to be directly coupled with the membrane curvature, in contrast, the activity of PKC does not have a direct relationship with the curvature strain and enzymatic activity [41]. The activity of PKC is instead modulated by nonlamellar-forming lipids via a less direct mechanism.

#### 2.3.2. Transporters

Liposomes have been commonly used for reconstituting different types of transporters to allow for the free diffusion of solution or catalysis of obligatory co-transporters. A large number of functional membrane proteins have been successfully reconstituted into liposomes but only a few examples will be discussed here. The reconstitution of colicin Ia and E1 in either soybean phospholipids or *E. coli* phospholipids show that there is channel formation in the liposomes but there are unspecific channels allowing passage of ions, such as rubidium, sodium, chlorine, potassium or phosphate but not of sugars [7,44]. An example of the reconstitution of selective transport comes from the d-glucose transporter, purified from human erythrocytes and extracted from detergents followed by incorporation into proteoliposomes. With incorporation of the d-glucose transporter, the proteoliposomes become permeable to d-glucose but not to l-glucose. The transport was inhibited by cytochalasin B which is a potent inhibitor of d-glucose transporter [45,46].

Several types of ATP-dependent ion transporters such as Ca^2+^/Mg^2+^-ATPase, Na^+^/K^+^-ATPase, and H^+^/K^+^-ATPase have been reconstituted into proteoliposomes [47]. Upon addition of ATP, ions are observed to be transported inwards and can form a complex. The single-channel property of channels incorporated into proteoliposomes can be investigated using the well-known patch-clamp method [48]. Channel activity is monitored following excision of the patch from the proteoliposomes. Ion-channel reconstitution makes possible the investigation of the influence of membrane lipid composition on channel function. The kinetic investigation of these channels under physiological conditions has been discussed elsewhere [47].

Another up-to-date method is using organic solvent or oil mixed with water that creates water-in-oil (W/O) microdroplets coated by phospholipid. The hydrophilic head group immerses in the water and the hydrophobic tail locates in the oil/organic solvent phase. The application of the water-in-oil system could cover a wide range of applications from monolayer, planer lipid bilayer and liposomes. Funakoshi *et al.* [49] and Maglia *et al.* [50] used a planer lipid bilayer formed by two microdroplets driven to come in contact to reconstitute ion channels in the bilayer. This method is extremely simple and reproducible. Recently, the water-in-oil microdroplets are extended to form liposomes by using droplet-transfer method invented by Yoshikawa [51]. By using this approach, it is possible to modulate the lipid compositions of outer and inner leaflets and furthermore to orient a reconstituted membrane protein in liposomes [52].

### 2.4. Nanodiscs

Nanodiscs offer a solution to some of the challenges described in the previous sections. The first attempt to reconstitute membrane proteins in the phospholipid bilayers using nanodisc technology was initiated by Sligar’s group a decade ago [8]. The nanodisc is a self-assembly of phospholipids and a membrane scaffold protein derived from human serum apolipoprotein A1. The detergent, cholate, can be used to solubilize phospholipids and membrane scaffold proteins into a micelle mixture. Following detergent removal with dialysis or Bio-beads adsorbent, a nanodisc self-assembles. The phospholipid associates as a bilayer domain while the membrane scaffold protein wraps around the edges of the discoidal structure in a belt-like configuration (Figure 1D). It is possible to modify the diameter of the bilayer disc by genetically engineering the apolipoprotein A1 by changing the number of amphipathic helices. By this approach, the diameter of nanodiscs can be made anywhere from 9.8 to 17 nm, and therefore accommodate a range of membrane proteins. The ratio of phospholipid: membrane scaffold protein is precisely defined which helps engineer the different size of membrane proteins in the nanodiscs. Detailed formation of different types of nanodiscs has been described elsewhere [53,54].

The great advantage of using nanodiscs is keeping the membrane proteins in aqueous solution, in native-like phospholipid bilayer environment that is soluble, stable, monodisperse and detergent-free. Most important, it isolates proteins or complexes as individual particles in monomeric or oligomeric states for analysis by techniques that range from activity assays to electron microscopy. Since 2003, there have been more than 100 membrane proteins reconstituted into nanodiscs [54], ranging from signaling receptors to transport machines. We will discuss the applications separately below.

#### 2.4.1. G Protein Coupled Receptors [55]

Nanodiscs have been used to analyze the influence of binding substrate on monodisperse receptors which are isolated from the cell-surface membrane. Those receptors include G protein-coupled receptors (GPCR) [55,56], cholera toxin receptor ganglioside G_M1_, bacterial chemoreceptor [57] and epidermal growth factor receptor. Introduced into nanodiscs, the receptors stay in monodispersed, controllable, predefined oligomeric states in which it is possible to characterize the oligomeric status. For example, two different GPCR proteins, the beta-adrenergic receptor (β_2_AR) and rhodopsin [58] have been extensively studied using nanodiscs. β_2_AR was one of the first receptors assembled into nanodiscs which was found to be functionally active (54% of starting activity recovered) and shown coupling to its G-protein. Rhodopsin is a light-activated GPCR present in the photoreceptor cells of the retina and transducin is an important G-protein naturally expressed in retina rods and cones. Assembly of functional rhodopsin into nanodiscs was found to activate transducin with high efficiency and to isolate the high affinity of transducin–metharhodopsin II complex. This provides strong evidence that the monomeric state of rhodopsin can activate and interact with the transducin. A dimeric rhodopsin nanodisc was separated for monomeric forms using sucrose density gradients. Even with two rhodopsins in the nanodiscs, interaction with a single transducin molecule was observed and found to activate the transductin with high efficiency [56].

#### 2.4.2. Enzymatic Activities, Cytochrome P450 and Its Ligand Binding

Numerous membrane associated enzymes have been incorporated into nanodiscs. Cytochrome P450 (CYP) enzymes have been extensively studied, including CYP2B4 [59], CYP6B1 [8], CYP73A5 [60] and CYP19 [61,62]. This system has provided a means for studying the extensive collection of membrane bound cytochromes P450 with the same biochemical and biophysical tools that have been previously limited to use with the soluble P450s. For example, the cytochrome P450 3A4 (CYP3A4) is a membrane-bound protein which is a human hepatic drug-metabolizing enzyme. Most studies of the ligand binding by CYP34A are carried out in the presence of detergents below their critical micelle concentrations [63,64] but are compared by the propensity of CYP34A to aggregate. Even in studies attempting to use liposomes, CYP3A4 is unlikely to exist in its native state because the detergent concentrations are much higher than the phospholipid concentrations. As a result, the understanding of the structure and composition of CYP3A4 in the lipid phase was limited and the membrane effect on CYP3A4 ligand binding behavior is unclear. Nanodiscs have been utilized to study CYP3A4 which displays monophasic reduction kinetics. With a high lipid–protein ratio, CYP3A4 is captured as a monomer. However, at lower lipid ratio, CY3A4 self-associates and heterogeneous behaviors are induced. The nanodiscs prohibit self-association in this case as there is only one CYP3A4 per nanodisc and show significant improvement in homogeneity and stability. This opens up new possibilities for detailed analysis of equilibrium and steady-state kinetic characteristics of catalytic mechanisms of CYP enzymes [63].

#### 2.4.3. Transporters and Channels

The Sec translocon is a membrane-embedded protein assembly that drives protein translocation into or across membranes. The core translocon is formed from a trimeric arrangement of SecY, SecE and SecG [65]. The SecYEG promoter has 15 transmembrane helices sitting in the phospholipid membrane. The oligomerization of SecYEG has been proposed to be necessary to proper function. Researchers were successful in reconstituting Sec into membrane vesicles in 1990 and have had great success in characterizing several partial reactions of SecYEG functions [65]. Reconstituting a single SecYEG into a nanodisc with different types of lipids [66] suggests that the acidic lipids can stabilize the SecYEG channel in the nanodisc bilayer and trigger dissociation of the SecA dimer. A model has been proposed by Alami *et al.* [66], suggesting that the dissociation of the SecA dimer provoked by the SecYEG complex is followed by activation of the SecA ATPase. Furthermore, Dalal *et al.* [67], using the nanodisc technology, have also shown that only the SecY dimer together with acidic lipids supports the activation of the SecA translocation ATPase. Recently, a high resolution single-particle Cryo-EM structure of single SecYEG complexes in nanodiscs, bound to translating ribosomes was first solved at subnanometer resolution [68]. It allows the SecYEG complex to be investigated in a natural lipid bilayer environment and identifies the ribosome-lipid interactions. Wu *et al.* [69] also used surface plasmon resonance to investigate the competitive binding of ribosomes and SecA. The data suggest that both ribosomes and SecA can interact simultaneously with SecYEG complex during membrane protein insertion, but SecA competes with ribosome when it binds to the SecYEG complex.

## 3. Comparisons

In the previous section, we have shown that membrane proteins can be assembled into four different types of model membrane and the activities of some of the membrane proteins can be retained, allowing their physicochemical properties to be studied. But is there a model membrane system that is the best for membrane-protein reconstitution? The reconstitution of the same membrane protein into different model membranes has been compared and, in this section, we list four membrane proteins with varying activities in different model membranes.

### 3.1. Ganglioside G_M1_ Receptors Binding Activity

Ganglioside G_M1_ is a naturally occurring native receptor that binds to cholera toxin via hydrogen bonds [70]. It is an excellent receptor for studying lipid-receptor interaction. Several different approaches to reconstituting the glycolipid receptor G_M1_ in model membranes have enabled the measurement of binding of its interaction partner cholera toxin. In liposomes and supported lipid bilayer systems, the ganglioside G_M1_ is free to diffuse across long distances and exhibits a non-uniform lateral distribution, *i.e.*, self-aggregation, even at low incorporation ratios. Therefore the binding activity of ganglioside G_M1_ with cholera toxin B is restricted [71]. Investigations of ganglioside G_M1_ incorporated into nanodiscs found reduced protein aggregation. Bricarello *et al.* [72] found that the reconstitution of a low concentration of ganglioside G_M1_ in nanodiscs, shows binding of cholera toxin with a significantly higher affinity than in liposomes or supported lipid bilayers. This is due to the interaction of ganglioside G_M1_ with the headgroup region of the disc which reduces the oligomerization, thereby causing a potential effect on the affinity of toxin binding. Thus, nanodisc technology restricts the ganglioside G_M1_ oligomerization by controlling the number of ganglioside G_M1_ monomer isolated by each nanodisc.

**Figure 4:**
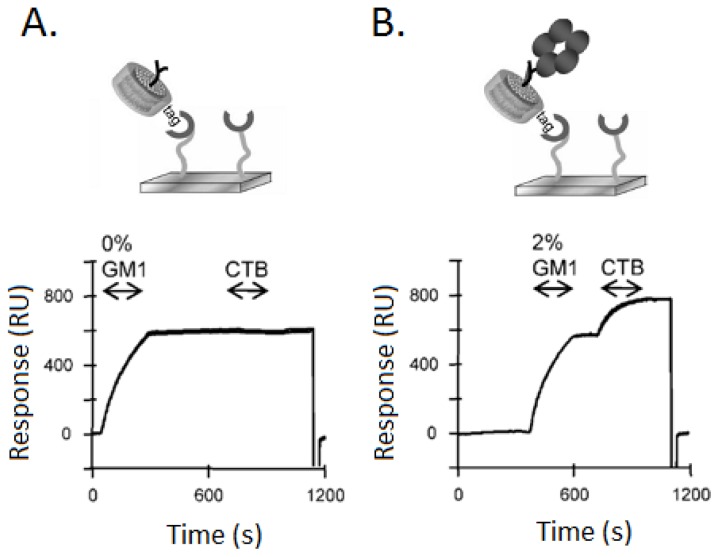
Capture of His-tagged nanodiscs to Ni-NTA sensor chips followed by binding of 20 nM cholera toxin subunit B (CTB) to nanodiscs that contain (**A**) 0% GM1 and (**B**) 2% GM1. The response measured in resonance units (RU) is linearly dependent on the mass bound to the sensorchip [73].

Borch *et al.* [73] have also used sensor chip-based surface plasmon resonance (SPR) technology to measure the detailed kinetic binding of the interaction between soluble molecules and membrane receptors inserted in the bilayer of nanodiscs. The corresponding SPR sensorgrams are displayed in Figure 4. Overall, the change of the sensorgram indicates that the SPR sensorchip is immobilized with histidine-modified nanodisc or the cholera toxin B bound to the nanodiscs. The sensorgrams in both Figure 4A,B shows the binding of nanodiscs (576 RU) on the antibody immobilization surface on the sensor chip. By injecting the cholera toxin B over two flow cells presented in Figure 4A and 4B, the SPR sensorgrams can detect the interaction of the cholera toxin B with Nanodisc with or without the existence of G_M1_. It has been revealed that the captured 2% G_M1_-nanodiscs bound 238 RU of the cholera toxin B without binding to the capturing nanodiscs without G_M1_ (Figure 4B). The measured kinetic values of the interaction are in agreement with those reported by previous studies on the interaction of the cholera toxin with the G_M1_ receptor embedded in different membrane systems. This, therefore, serves as a proof of concept that nanodiscs can be employed in kinetic SPR studies.

### 3.2. Liver Nuclear Ionic Channels

The nucleus envelope is composed of two bilayers (the outer nuclear membrane and inner nuclear membrane) and contains abundant ion channels, through which ions and small molecules diffuse between the cytoplasm, nucleoplasm and perinuclear (*i.e.*, intermembrane) space. The nuclear ionic channels represent a ubiquitous structure in the nuclei in a wide range of cells, although little is known about its functional properties. To characterize nuclear ionic channels, Guihard *et al.* [74] attempted to reconstitute nuclear envelope vesicles derived from the canine liver nuclei into a planar lipid bilayer and giant proteoliposomes. They found that the success rate of nuclear envelope fusion into planar lipid bilayers was extremely low although cardiac nuclear ionic channels were successfully incorporated into planar lipid bilayers. The detection of the nuclear ionic channels activity was not possible. Such a low efficiency can be explained by the clustering of nuclear envelope vesicles, and the low density of single vesicles, as well as the presence of residual chromatin and/or nuclear proteins (histones or lamins) which would prevent fusion events with the bilayer.

**Figure 5:**
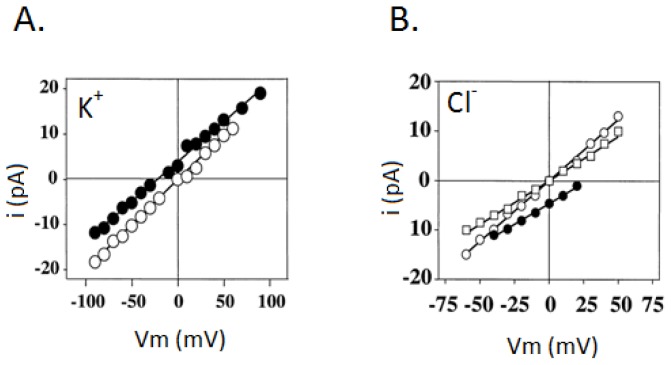
Current–voltage relationship curves of (**A**) the intermediate K^+^-selective nuclear ionic channels in asymmetrical 150/50 mM (•) KCl and in symmetrical 150/150 mM (○) KCl conditions and (**B**) Cl^−^-selective nuclear ionic channels in asymmetrical 150/50 mM (○) KCl and in symmetrical 50/50 mM (□) or 150/150 mM (•) KCl conditions [74].

Another approach is reconstituting nuclear envelope vesicles into giant proteolipsosmes and detecting the single ion channel by the patch-clamp technique [49]. Large conductance, voltage-gated, K^+^ and Cl^−^ selective nuclear ionic channels are characterized and plotted as a current–voltage relationship presented in Figure 5A,B respectively. It has been found that under asymmetrical 150/50 mM KCl conditions, the zero current potential for unitary currents is at 322 mV (Figure 5A). Calculated from the Goldman–Hodgkin–Katz (GHK) flux equation, a P_K_+/P_Cl_− ratio is 9.4. This value indicates the K^+^ selectivity for this channel. In Figure 5B, the Cl^−^ selective nuclear ionic channel yields a positive zero current potential of +27.3 mV, with a P_Cl_−/P_K_+ ratio of 80, indicative of a high Cl^−^ selectivity over K^+^. This suggests super fusion of the channel under asymmetrical (150/50 mM) KCl conditions. The current–voltage relationship curves indicate that the nuclear ion channels can be functionally characterized by incorporating the proteins into the giant proteoliposomes where it is possible to retain their channel activity. Furthermore, the measured activities are consistent with those described for native nuclear ion channels.

### 3.3. ATPase Activity of the P-Glycoprotein Transporter

P-glycoprotein, the most extensively studied ATP-binding cassette transporter, has been implicated in the phenomenon of multidrug-resistance in tumor cells and has been suggested to play a significant role in drug absorption and deposition. How P-glycoprotein interacts with its substrates is still unknown. Functional studies are limited because of the difficulty of obtaining large quantities of stable P-glycoprotein. Besides that, no ATPase activity of P-glycoprotein solubilized in detergent could be detected. When P-glycoprotein is reconstituted into proteolipsomes, it has detectable ATPase activity; however, the whole complex is very unstable. Heikal *et al.* [75] have further found that P-glycoprotein reconstituted in the proteoliposomes has a half-life of less than one day.

**Figure 6:**
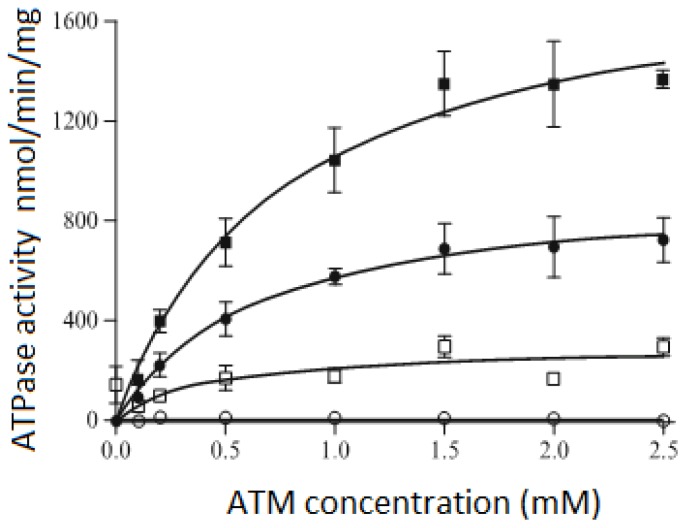
Comparsions of the ATPase activity of P-glycoprotein in nanodiscs (square) and proteoliposomes (circle). Open symbols: basal activity in the absence of drug; filled-in symbols: activity in the presence of nicardipine [76].

In 2009, Ritchie *et al.* [76] performed a detailed study of drug-stimulated ATPase kinase activity of P-glycoprotein using the nanodisc technology. The P-glycoprotein protein was reconstituted into both MSP1E3D1 disc and liposomes in order to compare its ATPase kinase activities. The results described in Figure 6 demonstrate that P-glycoprotein is functionally active when reconstituted into the nanodiscs (close squares). Comparing to the ATPase kinase activity of P-glycoprotein reconstitution in lipsosomes (close circles), there is a twofold increase in the maximum ATPase activity in the nanodiscs. This could be due to the uniform orientations of P-glycoprotein in the nanodiscs while there are two possible orientations in liposomes. These data not only show that P-glycoprotein is functionally active when reconstituted into the nanodiscs, but that it also exhibits higher specific activity than the current standard reconstitution system.

### 3.4. ATPase Activity of the MalFGK2 Complex in Nanodiscs, Detergents and Proteoliposomes

**Figure 7:**
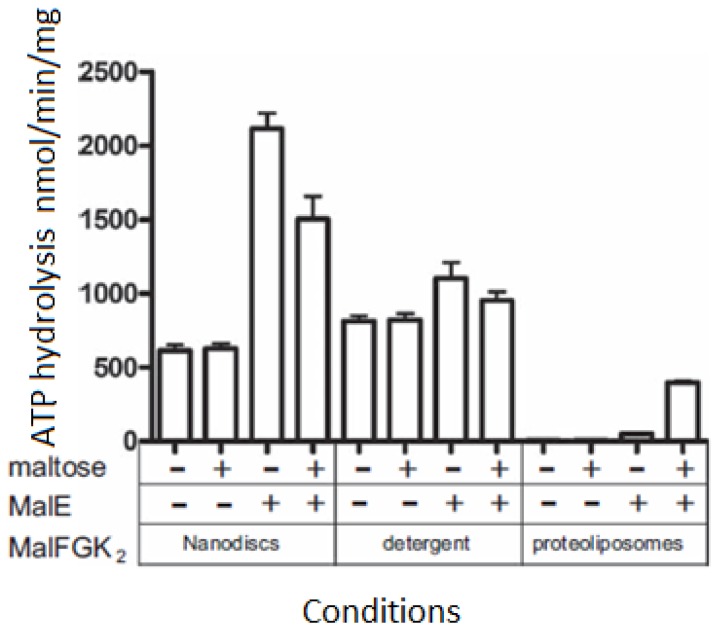
The ATPase activity of MalFGK2 was measured in nanodisc, detergent solubilized conditions and proteoliposomes in the presence of MalE or maltose [78].

ATP-binding cassette transporters utilize the energy of ATP hydrolysis to transport a wide range of substrates across cellular membranes and for non-transport-related processes such as translation of RNA and DNA repair [77]. A member of the ATP-binding cassette super family, the maltose transporter MalFGK2 from *E. coli*, together with the substrate-binding protein MalE, is one of the best-characterized ATP-binding cassette binding cassette transporters suitable for various reconstitution techniques. Bao and Fuong have reported the reconstitution of the maltose transporter in nanodiscs, in detergent and in proteoliposomes. The ATPase activity of the MalFGK2 complex in various environments is shown in Figure 7. The data presented in the first column of Figure 7 show that the basal ATPase activity for assembly in the nanodiscs and detergent (~700 nmol/min/mg) is 10-fold higher than in proteoliposomes because of the decrease in the activation energy barrier of the transporter [78] in detergent micelles and nanodiscs. However, in the presence of MalE, the rate of ATP hydrolysis increases in all assembly conditions. This is because MalE captures maltose and delivers the sugar to the transporter. Note that the basal ATPase activity assembly in the nanodiscs dramatically increases from 700 to 2300 nmol/min/mg. The maltose alone has no effect on the basal ATPase activity in the nanodiscs and detergent. However, in nanodisc and detergent, an inhibition of the ATPase activity was observed in the presence of both maltose and MalE in the nanodiscs. This is because that maltose reduces the binding affinity of the MalE–MalFGK_2_ complex, which therefore has reduced the ATPase activity. In proteoliposomes, the ATPase activity (~40 nmol/min/mg) shows a further 10-fold increase in the presence of both maltose and MelE in the figure. The author used another type of MalE mutant which binds maltose with higher affinity. This MalE mutant, in contrast, shows a reduction of the ATPase activity in proteoliposomes which has the same effect as the nanodiscs and detergents. Overall, proteoliposomes have shown a low basal ATPase activity because the lipid stabilized the transporter. However, the nanodiscs have been shown to be a better medium than proteoliposomes for studying the ATP hydrolysis ability of ATP-binding cassette transporters.

## 4. Conclusions

This review summarizes and compares the most up-to-date methods for reconstituting membrane proteins into model membranes. There is no superior method for reconstituting membrane proteins in the model membrane; instead two or more model membranes should be considered, depending on the particular needs of the system and the proteins of interest. In general, systems based on lipid bilayers supported on a solid substrate are still the most favored and well-developed of the methods to study membrane proteins in the bilayer. This approach allows detailed study of the fundamental properties of biological membranes and is practical to reproduce the bilayer system. On the other hand, the proteoliposome is more suitable for ion channel reconstitution in the bilayer, as well as for combination with the patch-clamp method to detect the ionic selectivity of the channel. Finally the self-assembled nanodiscs system provides a robust and common means for rendering these targets soluble in aqueous media while providing a native-like bilayer environment that maintains functional activities. Nanodisc technology offers another way to prepare monodisperse samples of membrane proteins in the bilayer environment, and it is emerging as the favored approach in studies concerning membrane protein complexes.
